# The Influence of Epigenetic Modifications on Metabolic Changes in White Adipose Tissue and Liver and Their Potential Impact in Exercise

**DOI:** 10.3389/fphys.2021.686270

**Published:** 2021-08-26

**Authors:** Jessie E. Axsom, Heath D. Schmidt, Lea Ann Matura, Joseph R. Libonati

**Affiliations:** ^1^Department of Biobehavioral Health Sciences, School of Nursing, University of Pennsylvania, Philadelphia, PA, United States; ^2^Penn Cardiovascular Institute, Smilow Translational Research Center, Perelman School of Medicine, University of Pennsylvania, Philadelphia, PA, United States; ^3^Department of Psychiatry, Perelman School of Medicine, University of Pennsylvania, Philadelphia, PA, United States

**Keywords:** liver, white adipose tissue, epigenetics, gluconeogcncsis, lipolysis, fatty acid oxidation

## Abstract

**Background:** Epigenetic marks are responsive to a wide variety of environmental stimuli and serve as important mediators for gene transcription. A number of chromatin modifying enzymes orchestrate epigenetic responses to environmental stimuli, with a growing body of research examining how changes in metabolic substrates or co-factors alter epigenetic modifications.

**Scope of Review:** Here, we provide a systematic review of existing evidence of metabolism-related epigenetic changes in white adipose tissue (WAT) and the liver and generate secondary hypotheses on how exercise may impact metabolism-related epigenetic marks in these tissues.

**Major Conclusions:** Epigenetic changes contribute to the complex transcriptional responses associated with WAT lipolysis, hepatic *de novo* lipogenesis, and hepatic gluconeogenesis. While these metabolic responses may hypothetically be altered with acute and chronic exercise, direct testing is needed.

## Introduction

**Figure 1:**
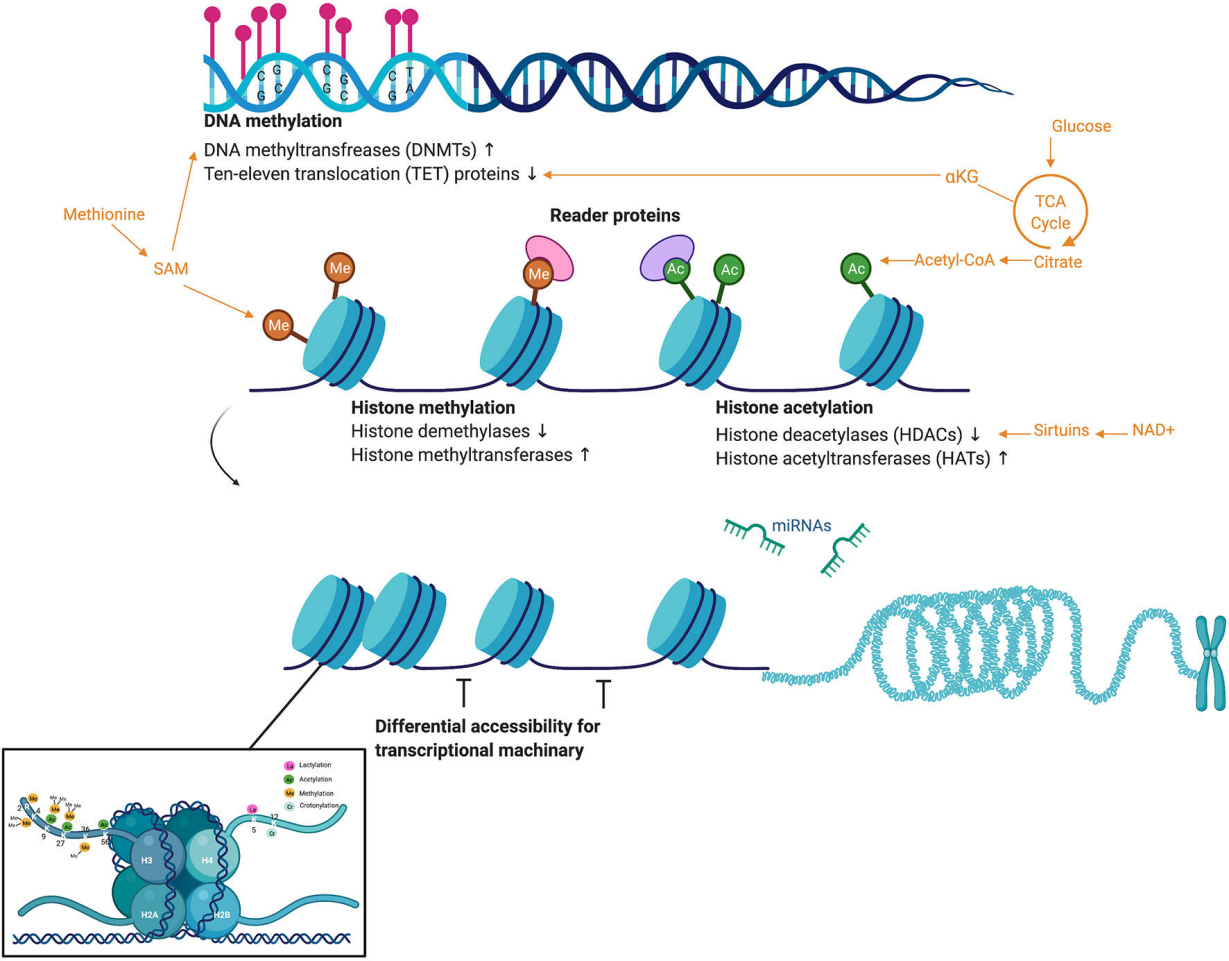
Overview of common epigenetic modifications, the activity of chromatin modifying enzymes, and the metabolites that act as substrate. DNA methylation most commonly occurs in regions of CpG nucleotides called “CpG islands.” DNA methylation is moderated by the chromatin modifying enzymes DNA methyltransferases (DNMTs), which deposit methylation marks and use methionine metabolism as a substrate, and ten–eleven translocation (TET) proteins, which mediate demethylation and use the TCA cycle intermediate alpha-ketoglutarate as a substrate. Once DNA is wound around histone proteins, chromatin modifying enzymes regulate a suite of histone modifications such as methylation (again using methionine metabolism) and acetylation (using acetyl-CoA). Sirtuins, a family of signaling proteins often involved in metabolic regulation, can act as NAD+ dependent deacetylases of both histones and non-histone proteins. While microRNAs are not considered canonical epigenetic modifications, they can affect the chromatin modifying enzymes depositing epigenetic marks, as well as recruit protein complexes that alter chromatin structure. These epigenetic marks and microRNAs affect differential accessibility of chromatin for transcriptional machinery. This figure created with Biorender.com.

Epigenetics integrates genetic, lifestyle, and environmental factors. Epigenetic modifications regulate gene expression without altering the underlying nucleotide sequence of the genome. Structurally, DNA is wrapped around eight histone proteins to form nucleosomes. Repeated nucleosomes form a “beads on a string” structural arrangement that is condensed into chromatin fibers and densely packaged into chromosomes (Figure 1). Epigenetic alterations alter the structure and accessibility of DNA through modifications to histone proteins as well as the DNA itself (Figure 1) (Portela and Esteller, 2010). The most well-characterized epigenetic marks are cytosine methylation and methylation and acetylation of the amino acid residues of the histone proteins (Portela and Esteller, 2010). While microRNAs are not considered canonical epigenetic modifications, they can affect chromatin modifying enzymes depositing epigenetic marks, as well as recruit protein complexes that alter chromatin structure (Moutinho and Esteller, 2017). Thus, epigenetic modifications and mechanisms that regulate these epigenetic marks themselves, including microRNAs, are regularly incorporated into the “epigenome.”

While it was previously thought that epigenetic marks were relatively stable after completion of embryonic development, especially the energetic process of methylation; it is now accepted that epigenetic marks are dynamic and responsive to a wide variety of stimuli (Portela and Esteller, 2010) and are an important link between the environment and gene transcription. A better understanding of interactions between the environment cues and the genome may improve the prevention, diagnosis, and treatment of chronic diseases, such as type 2 diabetes mellitus (T2DM) and heart disease.

A number of chromatin modifying enzymes orchestrate epigenetic responses to environmental stimuli. These include histone acetyltransferases (HATs), histone deacetyltransferases (HDACs), and DNA methyltransferases (DNMTs) (Portela and Esteller, 2010). Importantly, the activity of chromatin modifying enzymes partially depends upon the availability of metabolic substrates or co-factors (Figure 1), such as acetyl-CoA supplying histone acetylation, S-adenosyl-L-methionine supplying methylation, and NAD+-dependent Class III HDAC activity (Sirtuin I-7) (McGee and Hargreaves, 2019). There is a growing body of research examining how changes in these metabolic substrates or co-factors alter epigenetic modifications and subsequently gene transcription, cellular processes, and physiological and behavioral responses.

The majority of studies examining metabolic-epigenetic interactions have focused on pathological changes in substrate flux, most notably in the cancer metabolism field (Rinaldi et al., 2018). However, changes in substrate flux in a health-promotion context (i.e., exercise) may also be of interest. It is theoretically feasible that substrate flux during exercise may influence metabolite and co-factor availability for epigenetic alterations. This is of significant interest given exercise induces a myriad of epigenetic changes (Denham, 2018; Axsom and Libonati, 2019); an effect principally shown in skeletal muscle secondary to its responsiveness to exercise and the ease of obtaining samples via biopsy (Howlett and McGee, 2016; McGee and Walder, 2017; McGee and Hargreaves, 2019). There is a gap, however, in our understanding of how exercise influences epigenetics in two other important metabolic tissues—adipose tissue and liver. Currently, there are a very limited number of studies actually examining metabolism during exercise and epigenetic changes in adipose tissue and liver, making a review of this literature premature. However, there is a larger body of evidence examining metabolism-related epigenetic changes in the liver and adipose tissue.

Thus, the objective of this systematic review is to identify the current evidence for metabolism-related epigenetic changes in the liver and adipose tissue. Secondarily, this systematic review will also generate potential hypotheses relating exercise metabolism to epigenetic marks in these tissues.

## Manuscript

### Methods

**Figure 2:**
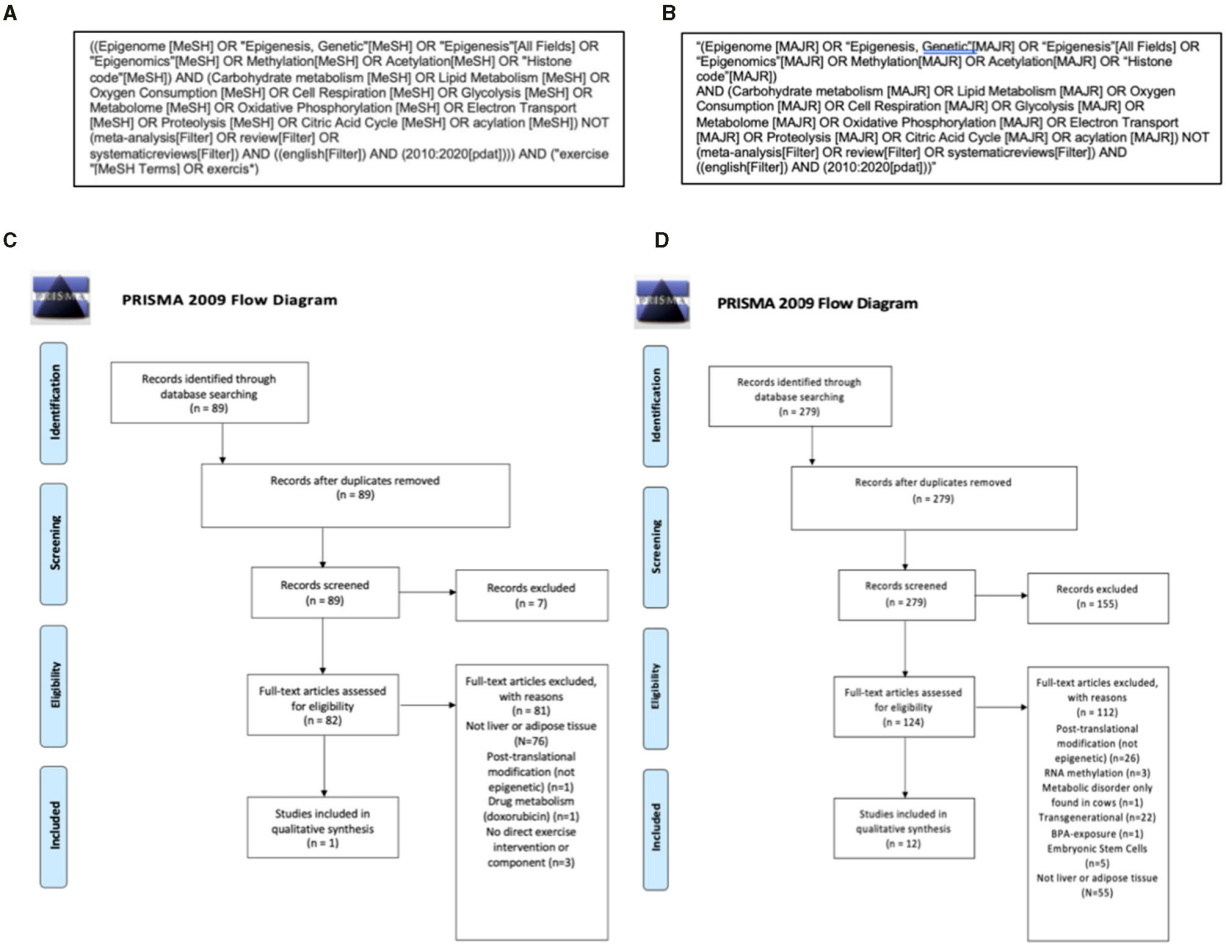
Search strategy and Preferred Reporting Items for Systematic Reviews and Meta-Analyses (PRISMA) flow diagrams of information detailing the systematic review process. An initial PubMed search **(A)** identified 89, with no duplicates and completed within the last 10 years. There were 82 articles that met the following criteria: original research in the English language with full-text availability that discussed exercise, measures of both cellular metabolism and epigenetic modifications and used human, animal (mammal), or cell line models. After a more thorough screening, 82 articles were excluded for the reasons listed below and one article was included for qualitative synthesis **(C)**. To increase the number of articles for qualitative synthesis, as well as identify novel applications of current literature to exercise physiology, a second Pubmed search was completed that did not require an exercise component in the studies **(B)**. This search yielded 279 articles and after two screenings there were 12 additional articles included for qualitative synthesis **(D)**. Between the two searches there were 13 final articles included for discussion.

A systematic review was conducted to assess current evidence for exercise-induced epigenetic modifications related to metabolism in adipose tissue and the liver. The PICOS research question was as follows—Population: Adipose tissue and the liver, Intervention: Exercise, Comparators: Sedentary, Outcome: Epigenetic marks related to metabolism, Study design: original research. Initial inclusion and exclusion criteria were established to ensure results addressed the research question and provided high-quality evidence. A University research librarian was consulted to decide which databases to search and what search terms to use. PubMed was determined to be the proper database to search as this is an experimental biology question. The controlled PubMed vocabulary MeSH terms were used in increase reproducibility of the search. The MAJR filter was applied, which requires the search terms are a major component of the resulting articles, to increase relevancy of the search (Figure 2A).

This search produced 89 results. References and abstracts of each search result were exported to Mendeley, a citation manager software. First-pass abstract screening was completed using the following initial inclusion and exclusion criteria: original research in the English language with full-text availability that discussed exercise, measures of both cellular metabolism and epigenetic modifications, and used human, animal (mammal), or cell line models. No duplicate results were included, and research had to be published within the past 10 years to ensure up-to-date evidence. After the initial screening, there were 82 articles that subsequently underwent a second screening using the following additional criteria: (a) relevance to adipose tissue or the liver, (b) exclusion of non-epigenetic post-translational modifications, and (c) exclusion of metabolism of a drug that was not an exercise-mimetic (Figure 2C). The second screening yielded only one paper. This is an expected limitation given the nascence of this area of study (see section Limitations). This sample size was not sufficiently large enough to conduct a systematic review. To address this, we completed a second search detailed below.

A second search was completed using the same inclusion and exclusion criteria without requiring an exercise component in the study (Figure 2B) and the following modified PICOS statement—Population: Adipose tissue and the liver, Intervention: Modification of metabolism, Comparison: No modification of metabolism, Outcome: Epigenetic changes, Study design: original research. This allowed for a more thorough understanding of the literature and the opportunity to discuss novel applications to exercise physiology. This expanded search yielded 279 results in PubMed. After initial screening there were 124 articles that then underwent a second, more thorough screening. The following additional criteria were used: (a) relevance to adipose tissue or the liver, (b) exclusion of non-epigenetic post-translational modifications, (c) exclusion of RNA methylation, (d) exclusion of intergenerational studies, (e) metabolism of a drug that was not an exercise-mimetic, (f) embryonic stem cell models, and (g) exclusion of a metabolic disorder that exclusively occurs in cows (Figure 2D). After the second screening, 12 results met these criteria. The two searches combined yielded 13 studies that met the inclusion criteria of original research available in English published between 2010 and 2020 examining metabolism-related epigenetic changes in liver and adipose tissue, with only one of the studies directly including an exercise component (Table 1).

**Table 1:** The 13 studies that met the inclusion criteria of original research available in English completed between 2010 and 2020 examining metabolism-modulated epigenetic changes in liver and adipose tissue.

**In text citation**	**Model**	**Metabolic measures**	**Epigenetic measures**	**Major outcome**
**REGULATION OF LIPOLYSIS**				
**REGULATION OF** ***DE NOVO*** **LIPOGENESIS**				
Castellano-Castillo et al., 2019	Human visceral adipose tissue samples	BMI, insulin resistance	Histone methylation	↑ H3K4me3 at promoter regions of genes involved in lipid metabolism and inflammation and positively correlated with BMI and insulin resistance
Bialesova et al., 2017	Human adipose tissue samples, adipocytes *in vitro*	Obesity, basal TAG lipolysis	DNA methylation	PLIN1 gene is regulated by methylation and methylation is inversely correlated with basal lipolysis
Das et al., 2015	Murine adipose tissue and liver samples, adipocytes *in vitro*	TAG lipolysis	microRNA activity	miR124-a regulates TAG lipolytic activity during fasting and re-feeding
Lane et al., 2019	Liver biopsies from NASH patients, Hepatocytes, HEK293T cells	DNL gene expression regulation and hepatic lipid homeostasis	Histone methylation	A glucose-induced protein modification triggers a multistep epigenetic pathway which ↑ transcription of lipogenic genes and DNL
Feng et al., 2011	Murine liver samples	DNL gene expression regulation and hepatic lipid homeostasis	Histone acetylation	HDAC3 modulates histone acetylation and gene expression in a circadian-rhythm dependent manner to represses DNL and maintain hepatic lipid homeostasis
Guo et al., 2017	Murine liver samples, murine primary hepatocytes	DNL enzyme activity and hepatic lipid homeostasis	microRNA activity	miR-212-5p binds to DNL enzymes to inhibit their activity. ↑ miR-212-5p ↓ DNL enzyme activity and was associated with ↓ hepatic TAG accumulation.
Goedeke et al., 2014	Murine liver samples	Plasma TAG levels, hepatic lipid homeostasis	microRNA activity	Long-term inhibition of miR-33 in mice fed a high fat diet ↑ expression of DNL genes, ↑ hepatic steatosis, ↑ hypertriglyceremia
Hahn et al., 2017	Murine liver samples	Hepatic lipidomics	DNA methylation	Dietary restriction (DR) protects against age-associated changes in genome-wide DNA methylation DR limited DNA methylation to gene regions involved in lipid metabolism, associated with ↓ DNL gene expression, ↓ hepatic TAG accumulation
**REGULATION OF HEPATIC GLUCONEOGENESIS**				
Ravnskjaer et al., 2013	Genetic mouse models, murine primary hepatocytes, HEK293T cells	Gluconeogenic gene regulation	Histone acetylation, histone methylation	Glucagon-induced activation of CREB and CRTC2 promoted recruitment KAT2B and a subunit of a histone methyltransferase complex that ↑ H3K9 acetylation and expression of gluconeogenic genes
Tsai et al., 2013	Ob/ob mouse model, murine primary hepatocytes, HEK293T cells	Gluconeogenic gene regulation	Histone methylation	Coactivator CRTC2 associates with PRMT5 to ↑ H3R2me and chromatin accessibility at CREB binding sites in gluconeogenic gene promoters
Sen et al., 2017	HepG2 cells	Gluconeogenic gene regulation	Histone methylation	TCF-19 preferentially interacts with H3K4me3 during high glucose conditions and is recruited to the promoter regions of gluconeogenic genes to transcription
Li et al., 2015	Hepatocytes (*in vitro* and *in vivo*)	Gluconeogenic gene regulation	microRNA activity	miR-214 represses gluconeogenesis via ↓ expression of ATF4 and ↓ transcription of gluconeogenic genes
**HEPATIC MITOCHONDRIAL FUNCTION, MORPHOLOGY, AND BIOENERGETICS**				
Ryu et al., 2014	SIRT7 KO mouse models	Liver mitochondrial metabolism	SIRT7 activity and histone deacetylation	Fasting conditions ↑ SIRT7 expression, ↓ GABPβ1 acetylation, and ↑ expression of oxidative phosphorylation genes in the liver SIRT7 KO mice had impaired exercise performance, attributed to cardiac dysfunction

*One study met the additional inclusion criteria of containing an exercise component (Ryu et al., 2014). Studies are listed in order they appear in the text and organized according to the metabolic process relevant to the results (shaded in gray)*.

### Results

The included studies are addressed below, with the following areas discussed: (1) evidence of epigenetic contributions to metabolic processes in white adipose tissue (WAT) (section Evidence of Epigenetic Changes Mediating Lipid Metabolism in White Adipose Tissue) and the liver (section Evidence of Epigenetic Changes Contributing to Lipid and Glucose Handling in the Liver) and (2) a discussion of potential hypotheses regarding how exercise may impact metabolism-related epigenetic marks in WAT and liver.

#### Evidence of Epigenetic Changes Mediating Lipid Metabolism in White Adipose Tissue

While WAT has a diverse range of metabolic functions, its primary role is energy storage. When excess fatty acids are consumed or produced, they are stored in the cytosolic lipid droplets of adipocytes as triacylglycerols (TAGs) (Lass et al., 2011). During states of energy demand (such as fasting or exercise), WAT is stimulated to breakdown TAGs through lipolysis. The three main enzymes that regulate the breakdown of TAGs in WAT are adipose triglyceride lipase (ATGL), hormone sensitive lipase, and monoglyceride lipase (Lass et al., 2011). Triacylglycerol lipolysis releases free fatty acids and glycerol to help provide energy to peripheral tissues. When there is an excess of WAT, however, this carefully regulated cycle of TAG storage and breakdown becomes dysregulated. Instead of stimulated lipolysis releasing much needed free fatty acids and glycerol, there is an increase in basal lipolysis and a rise in plasma free fatty acids even when peripheral tissues do not require them for energy (Bialesova et al., 2017). Elevated free fatty acids contribute to insulin resistance, the development of T2DM, and metabolic syndrome (Bialesova et al., 2017) and epigenetic changes have been postulated as one mechanism mediating the increase in basal lipolysis associated with excess WAT (Bialesova et al., 2017).

Several of the included studies in the systematic review examined associations between epigenetic marks in WAT and metabolic traits in humans (Table 1). In the first included study, Castellano-Castill et al. found that H3K4me3 was enriched at the promoter regions of genes involved in lipid metabolism and inflammation in human adipose tissue samples (Castellano-Castillo et al., 2019). These H3K4me3 marks were positively correlated with body mass index and insulin resistance of the human subjects (Castellano-Castillo et al., 2019), suggesting histone trimethylation is one epigenetic mechanism that contributes to the dysregulation of lipid metabolism and inflammation in poor metabolic health (Table 1). Lipolysis is regulated by several mechanisms, including beta-adrenergic stimulation, hormone signaling, and the activity of lipid droplet proteins (Lass et al., 2011). One such regulatory lipid droplet protein is perilipin-1, which is encoded by the *PLIN1* gene (Lass et al., 2011). In the basal state, perilipin-1 is unphosphorylated and bound to a co-activator of ATGL (one the aforementioned enzymes mediating TAG breakdown). Perilipin-1 thus acts as an important inhibitor of basal lipolysis in adipocytes (Lass et al., 2011). Upon stimulation, perilipin-1 becomes phosphorylated, the coactivator dissociates from perilipin-1 and activates ATGL, which promotes lipolysis (Lass et al., 2011). Obesity (broadly defined as excess WAT) is associated with decreased perilipin-1 protein levels and *PLIN1* mRNA in adipose tissue, but the regulatory mechanisms governing changes in *PLIN1* expression have not been well-elucidated (Bialesova et al., 2017). One possibility is DNA methylation, which has been associated with decreased gene expression. In the second included study, Bialesova et al. found adipocytes isolated from obese women have increased DNA methylation at the PLIN1 gene promoter compared to never-obese women (Table 1). This obesity-associated hypermethylation was inversely correlated with PLIN1 mRNA expression and basal lipolysis activity (Bialesova et al., 2017), suggesting obesity may incur an epigenetic change (hypermethylation) that decreases the expression of an important regulatory protein (perilipin-1), leading to increased levels of basal lipolysis. While this is purely correlative evidence, further experiments in cell culture showed that adipocytes containing a methylated PLIN1 promoter region exhibited decreased gene expression (Bialesova et al., 2017). When adipocytes were treated with a DNMT inhibitor, methylation of the *PLIN1* gene decreased, *PLIN1* mRNA increased, and perilipin-1 protein levels likewise increased (Bialesova et al., 2017). Thus, these cell culture experiments offer *in vitro* evidence of DNA methylation regulating perilipin-1 and subsequently ATGL activity and lipolysis.

Another included study investigated microRNA regulation of lipolysis in WAT. Das et al. found microRNA 124-a (miR124-a) regulates ATGL and one of its coactivators (Das et al., 2015) (Table 1). Ectopic expression of miR-124a in adipocytes led to reduced lipolytic activity and increased cellular TAG accumulation (Das et al., 2015). These effects were prevented, however, by overexpressing ATGL lacking the target site of miR124-a. Additionally, a strong negative correlation between miR-124a and ATGL in both mouse WAT and the liver have been shown. This negative correlation fluctuated with feeding status: high ATGL and low miR-124a levels during fasting and the opposite during feeding (Das et al., 2015). These results indicate that miR-124a may regulate the expression of ATGL and lipolytic activity, in a way that maintains metabolic flexibility (Das et al., 2015). Together, these experiments provide evidence for epigenetic modulation of lipolysis in WAT via regulating ATGL, which has important implications for overall metabolic health.

#### Discussion: How Might Epigenetic Changes Mediate White Adipose Tissue Response to Exercise?

**Figure 3:**
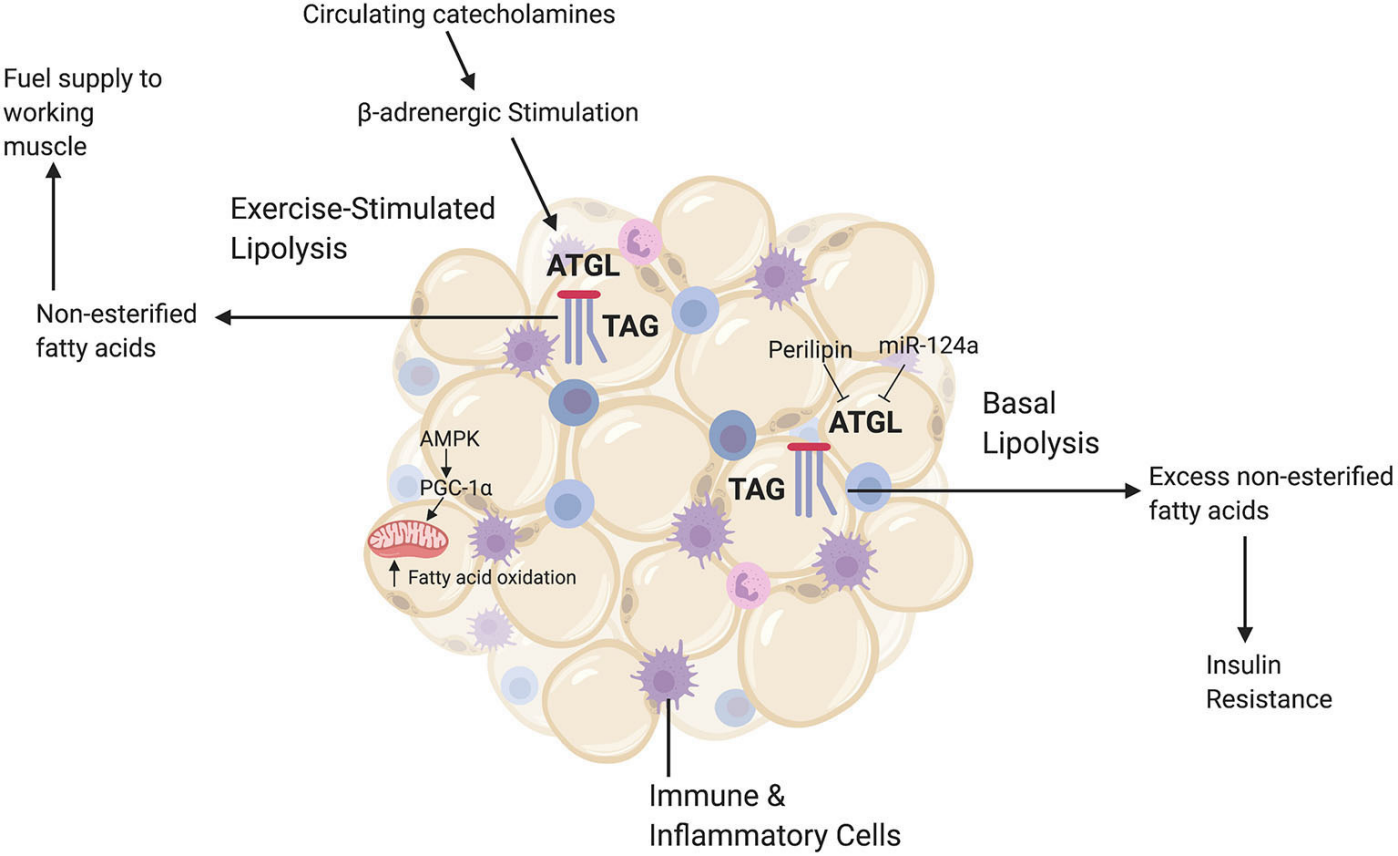
A brief summary of relevant white adipocyte tissue (WAT) functions. White adipocyte tissue has a diverse range of metabolic roles, including the release of signaling hormones and acting as a reservoir for immune and inflammatory cells. The main role of WAT, however, is energy storage. Excess fatty acids are stored in WAT as triacylglycerides (TAGs). When there are increased energy demands, such as during exercise, catecholamines trigger the catabolic enzyme ATGL to break down TAGs via lipolysis and release free fatty acids (or non-esterified fatty acids) to the circulation, which can then be used by skeletal muscle. Additionally, exercise upregulates the energy-sensing molecule AMPK and the master regulator of mitochondrial biogenesis pgc-1alpha. This upregulation increases fatty acid oxidation rates in adipocytes to meet increased energy demands. During basal conditions, however, energy demand is low and ATGL is inhibited by lipid droplet proteins such as perilipin-1 and microRNAs such as miR-124a. When there is an excess of WAT, this careful regulation of lipolysis becomes dysregulated and ATGL breaks down TAGs even when fatty acids are not needed by peripheral tissues. High levels of basal lipolysis lead to poor metabolic health outcomes such as insulin resistance. This figure created with Biorender.com.

The epigenetic impact of exercise on WAT metabolism may be important to consider given its implications for long-term metabolic health. White adipose tissue is central in catecholamine-induced lipolysis and mobilization of free fatty acids during acute exercise. This is significant because as exercise duration increases there is more reliance on fatty acid oxidation as a fuel source (Arner et al., 1990; Romijn et al., 1993; Brooks, 1997; Goodpaster and Sparks, 2017); with even low-intensity exercise increasing the catecholamine epinephrine three-fold above basal levels (McMurray et al., 1987; Henderson et al., 2007) (Figure 3). Along with catecholamines, the release of hormones such as growth hormone during exercise can induce regional increases in WAT lipolysis during exercise. The aforementioned enzyme ATGL, which helps initiate TAG catabolism, is important in mediating WAT-supplied FFA to skeletal muscle (Denham, 2018). We hypothesize that repeated exposure to catecholamine and hormone release during exercise might induce epigenetic changes in adipose tissue that target enzymes such as ATGL. It is plausible that these epigenetic changes could mediate the increased efficiency of exercise-stimulated lipolysis, which enhances exercise capacity and is a known adaptation in WAT to chronic exercise training (Ogasawara et al., 2015).

Fatty acid oxidation increases after an acute bout of exercise (Henderson et al., 2007) as well as an adaptation to chronic exercise training. Exercise training induces higher rates of fatty acid oxidation both at rest and during subsequent exercise (Van Loon et al., 1999). Exercise training increases adipocyte gene and protein expression of two key metabolic regulators: AMP-activated protein kinase (AMPK) and the previously mentioned transcriptional co-activator pgc-1alpha (Ruschke et al., 2010). Upregulation of AMPK and pgc-1alpha helps enhance fatty acid oxidation and mitochondrial biogenesis in adipose tissue, which helps decrease the need to transport excess fatty acids out of adipocytes via basal lipolysis (Ruschke et al., 2010) (Figure 3). Epigenetic changes in WAT that upregulate AMPK and pgc-1alpha could contribute to WATs enhanced ability with training to produce more energy and metabolic flexibility during the subsequent exercise session. It has been established that there are general, genome-wide epigenetic mark changes in WAT following exercise (Fabre et al., 2018). More targeted investigations could specifically examine whether these exercise-induced epigenetic changes in WAT target lipolysis enzymes (for example, ATGL, from the discussed studies) and fatty acid oxidation genes.

White adipose tissue supplies increased free fatty acid flux to skeletal muscle during exercise and exercise stimulates skeletal muscle lipolysis. Exercise induces changes in histone acetylation, DNA methylation, and microRNA activity in skeletal muscle (Denham, 2018). Many of these epigenetic changes target key metabolic genes, including pgc-1alpha (Barres et al., 2012; Denham, 2018). Fatty acids themselves are not known to be substrates for epigenetic marks. However, fatty acid oxidation can supply acetyl-CoA for histone acetylation and lipids can contribute large amounts of acetyl-CoA for histone acetylation (McDonnell et al., 2016). It is not known how changes in fatty acid flux, and the demand for fatty acids as a fuel source during exercise, might affect metabolite supply preference for histone acetylation. The results included examples of fatty acid exposure inducing changes in microRNA activity and DNA methylation in skeletal muscle. These epigenetic changes affected some of the same key transcriptional regulators of skeletal muscle fatty acid metabolism that have been shown to be epigenetically regulated with exercise, including pgc-1alpha. Clearly, there is an important relationship between fatty acid metabolism and epigenetic activity in skeletal muscle. The mechanisms driving lipid-associated epigenetic changes in skeletal muscle are not yet clear.

#### Evidence of Epigenetic Changes Contributing to Lipid and Glucose Handling in the Liver

The liver plays critical roles in energy production, energy storage, processing of metabolites such as free fatty acids, packaging and secretion of lipids, and detoxification of byproducts such as lactate. Given the metabolic flexibility demanded of the liver, it is reasonable to hypothesize epigenetic modifications may both modulate and be responsive to metabolic changes in the liver.

##### *De novo* Lipogenesis

**Figure 4:**
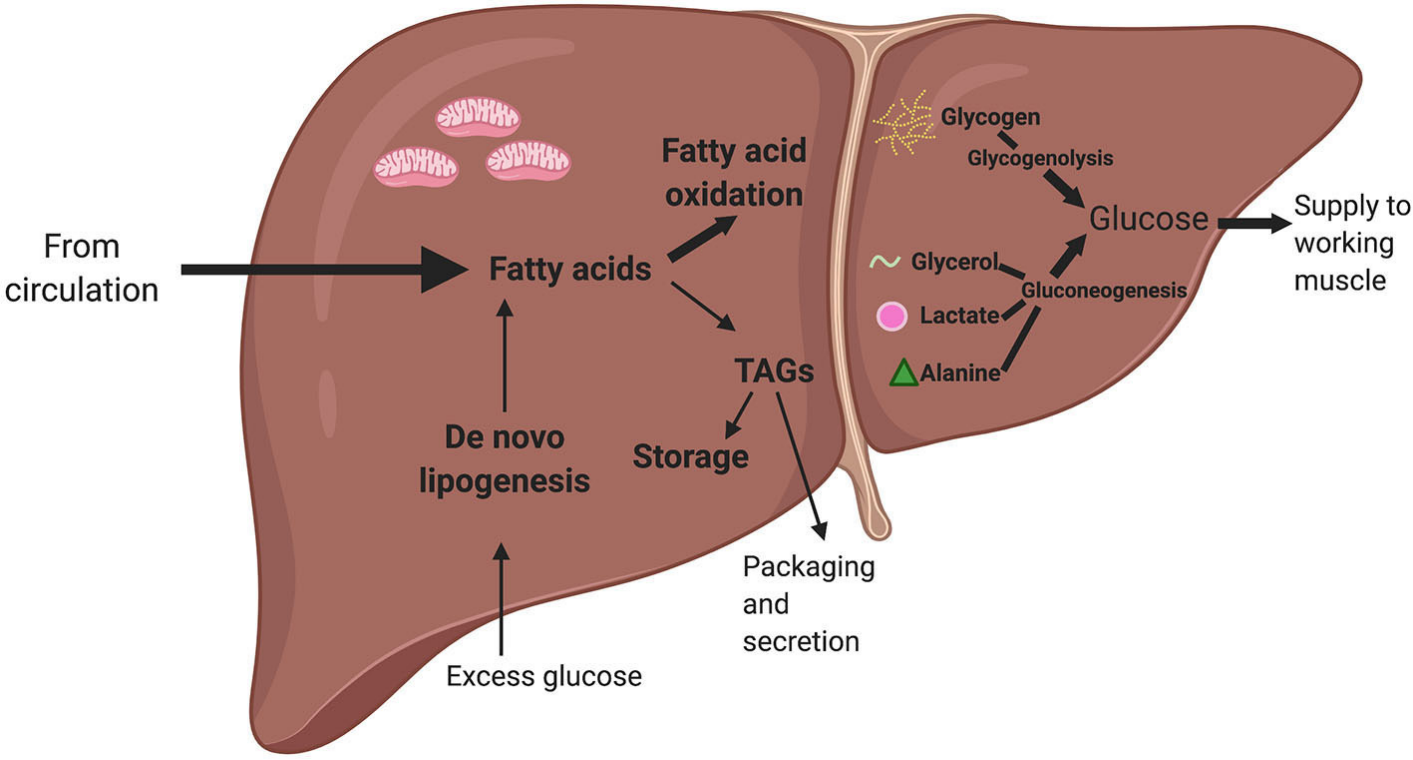
A simplified summary of liver lipid and glucose metabolism. Fatty acids from the circulation can either be used for fatty acid oxidation or converted into triacylglycerides (TAGs), which can either be stored or packed and secreted back to the circulation. When there is excess glucose in the circulation, the liver converts glucose into fatty acids via a process known as *de novo* lipogenesis. When there is a low level of glucose in the circulation, the pancreatic hormone glucagon triggers the liver to generate glucose. The liver can use glycerol (released by white adipose tissue via lipolysis), lactate (a metabolite produced via anaerobic glycolysis), and alanine (an amino acid) as precursors. The liver can also produce glucose by breaking down glycogen (or stored glucose) via a process called glycogenolysis. The glucose from both gluconeogenesis and glycogenolysis can then be released to working skeletal muscle during exercise. Bold arrows indicate metabolic processes most active during acute exercise. This figure created with Biorender.com.

During periods of high glucose consumption, when glycogen stores are high, the liver converts excess glucose to fatty acids and TAGs for storage, i.e., *de novo* lipogenesis (Ameer et al., 2014) (Figure 4). Hepatocyte glucose influx triggers a transcriptional response to increase lipogenic enzymes. Carbohydrate response element binding protein (ChREBP) is an important transcription factor mediating this glucose-induced *de novo* lipogenesis (Uyeda and Repa, 2006). It is unclear, however, exactly how glucose activates ChREBP. The fourth included study by Lane et al. (2019) used experiments in primary hepatocytes and HEK293T cells to show that regulatory protein (HCF-1) becomes O-GlcNAcetylated (a common protein modification) in response to glucose, and this glucose-induced protein modification then allows HCF-1 to bind to ChREBP (Table 1). Once bound, the HCF-1: ChREBP complex acts near the promoters of lipogenic genes. The presence of the complex is required for H3K4me3 (an epigenetic mark association with active transcription, Benayoun et al., 2014) and recruitment of an epigenetic activator in these lipogenic gene promoter regions (Lane et al., 2019). This mechanism is evidence of a glucose-induced multistep epigenetic pathway that promotes transcription of lipogenic genes and *de novo* lipogenesis.

Lipogenesis in the liver is also regulated by circadian rhythms, as are many metabolic processes (Feng et al., 2011). The transcriptome varies with circadian rhythms and these changes in gene expression are thought to be regulated, in part, by changes in histone acetylation. In the next included study, Feng et al. (2011) found the histone deacetylase 3 (HDAC3) is recruited to the genome in a circadian rhythm-dependent manner in the mouse liver (Table 1). Histone deacetylase 3 bound to over 14,000 sites on the genome when mice were resting (during the day) and only 120 sites when mice were active and feeding (at night) (Feng et al., 2011). Histone deacetylase 3 enzyme activity depends on its interactions with nuclear receptor corepressors, or proteins that inhibit gene expression. The circadian rhythm-associated transcriptional repressor Rev-erbα recruited HDAC3 to the genome. Histone deacetylase 3 and Rev-erbα were bound to the genome near genes that regulate lipid metabolism. Liver-specific deletion of HDAC3 or Rev-erbα resulted in a large increase in *de novo* lipogenesis and liver TAG content, indicating an accumulation of fat in the mouse liver (hepatic steatosis) (Feng et al., 2011). These results indicate that circadian rhythm-dependent activity of a histone modifying enzyme modulates histone acetylation and gene expression to repress *de novo* lipogenesis and maintain hepatic lipid homeostasis.

While *de novo* lipogenesis and stored TAGs can be an important energy source for the liver, dysregulated lipogenesis leads to adverse metabolic outcomes (Ameer et al., 2014). In fact, dysregulated *de novo* lipogenesis is the largest contributor to hepatic steatosis (Guo et al., 2017). Thus, there is great interest in studying dysregulated *de novo* lipogenesis and potential therapeutic targets. Two included studies investigated liver microRNA activity in the context of a high fat diet challenge (Table 1). In the first, Guo et al. (2017) demonstrated that microRNA-212-5p binds to two key enzymes for *de novo* lipogenesis, fatty acid synthase (FASN) and stearoyl-CoA desaturase-1 (SCD1), and inhibits their activity. Overexpression of miR-212-5p decreased protein levels of FASN and SCD1. Decreased expression of these two enzymes was associated with reduced TAG accumulation in both primary hepatocytes and mouse livers (Guo et al., 2017). Sterol regulatory element-binding proteins are transcription factors that regulate *de novo* lipogenesis. These transcription factors target expression some of the key aforementioned enzymes (including FASN). The second study, by Goedeke et al., established that long-term inhibition of microRNA-33 in mice fed a high fat diet increased expression of a transcriptional activator and downstream *de novo* lipogenesis genes. miR-33 inhibition during a high fat diet also caused hepatic steatosis and hypertriglyceremia compared to control animals with functional miR-33 (Goedeke et al., 2014). Both studies suggest microRNA could be novel therapies in non-alcoholic steatohepatitis (NASH). Non-alcoholic steatohepatitis, a type of non-alcoholic fatty liver disease (NAFLD), is characterized by hepatic steatosis, inflammation, and fibrosis, and currently has no FDA-approved pharmacological treatments, making microRNA therapy an appealing novel approach. One of the risk factors for NASH, in addition to high dietary intake of fats, is aging. Lipids accumulate in the liver in older age. This age-associated increase in hepatic lipids is accompanied by altered genome-wide DNA methylation (Hahn et al., 2017). The next included study (Hahn et al., 2017) implemented a dietary restriction intervention in mice during aging and found genome-wide patterns of DNA methylation were remodeled and age-associated methylation patterns were delayed. DNA methylation was instead targeted to genes involved in lipid metabolism and there was decreased gene expression of *de novo* lipogenesis related genes, accompanied by lower hepatic TAG content (Hahn et al., 2017).

##### Gluconeogenesis

In addition to *de novo* lipogenesis and regulating lipid homeostasis, one of the liver's most important roles is performing gluconeogenesis to produce glucose during energy-depleted conditions such as fasting. The expression of key gluconeogenic enzymes are controlled by complex transcriptional regulation that are activated by hormones and signaling molecules. Glucagon, a hormone that regulates blood glucose, increases gluconeogenic gene expression. Glucagon activates the cAMP pathway and phosphorylation of the transcription factor cAMP response element-binding protein (CREB). In the subsequent included study, experiments by Ravnskjaer et al. (2013) in mouse models, hepatocytes, and HEK293T cells revealed glucagon also dephosphorylates and activates CREB-regulated transcriptional coactivator 2 (CRTC2), a coactivator of CREB. Glucagon-induced activation of CREB and CRTC2 promoted recruitment of a chromatin modifying enzyme (lysine acetyltransferase 2B, KAT2B) and a subunit of a histone methyltransferase complex (WD repeat-containing protein 5, WDR5) (Ravnskjaer et al., 2013) (Table 1). This recruitment increased histone 3 lysine 9 acetylation, an epigenetic mark associated with active promoters, in gluconeogenic gene regions. Increased H3K9 acetylation was associated with increased expression of gluconeogenic genes (Ravnskjaer et al., 2013). The next included study, authored by the same group, found that coactivator CRTC2 associates with another chromatin modifying enzyme (arginine methyltransferase 5, PRMT5), to increase a different epigenetic mark (histone 3 arginine 2 methylation) and thus chromatin accessibility at CREB binding sites in gluconeogenic gene promoters (Tsai et al., 2013) (Table 1). Taken together, these studies indicate chromatin modifying enzymes respond to hormonal stimuli and regulate transcription factor-mediated gluconeogenic responses.

During high glucose conditions, gluconeogenesis is not energetically needed or favorable. Two included studies investigated regulatory mechanisms that work to suppress gluconeogenesis. In the first, Sen et al. (2017) found that when liver cells were exposed to high glucose, transcription factor 19 (TCF19) preferentially interacts with a specific epigenetic mark (H3K4me3, lysine 4 trimethylation on histone 3) (Table 1). Transcription factor 19 forms a complex with nucleosome-remodeling deacetylase (NuRD) components and is recruited to the promoter regions of gluconeogenic genes to repress their transcription (Sen et al., 2017). The second study (Li et al., 2015) showed evidence in hepatocytes of miR-214 repressing gluconeogenesis via reduced expression of the transcription factor activating transcription factor 4 (ATF4) and downstream transcriptional activity of gluconeogenic genes (Table 1). Thus, there is evidence of the epigenome both promoting transcription of gluconeogenic genes and repressing gluconeogenic genes as needed in response to environmental stimuli.

##### Liver Mitochondria

The final included study, by Ryu et al., investigates epigenetic regulation of liver mitochondria. Sirtuins are a family of regulatory proteins that can act as deacetylases and use NAD+ as a substrate. The nuclear Sirtuin SIRT7 deacetylates lysine residues on GABPβ1, an important regulator of nuclear-encoded mitochondrial genes that plays a crucial role in promoting mitochondrial adaptation to physiological challenges in the mouse liver (Ryu et al., 2014). SIRT7/GABPβ1 signaling promotes mitochondrial adaptations during fasting, feeding, and aging. During fasting, SIRT7 expression was increased, GABPβ1 acetylation was decreased, and expression of oxidative phosphorylation genes in the liver were increased (Ryu et al., 2014). Of note, this was the only study to directly include exercise in their data. *Sirt7* knock-out mice exhibited reduced exercise performance. The authors attributed this to the cardiac dysfunction (including pathological hypertrophy and reduced ejection fraction) associated with *Sirt7* knock-out mice, but further studies would be needed to confirm cardiac dysfunction is the sole cause of reduced endurance exercise capacity in *Sirt7* knock-out mice.

### Discussion: How Might Epigenetic Changes Mediate Liver Response to Exercise?

The liver is known to have several important key roles during exercise that require metabolic flexibility, which could be epigenetically regulated. The liver helps maintain energy supplies and buffer stress responses during exercise, replenish energy supplies after cessation of exercise, and modulate long-term adaptations to repeated exercise. Liver responses vary depending on the intensity, duration, frequency, and mode of exercise being performed, as well as nutritional intake. During acute exercise, there is rapid depletion of muscle glycogen and a large increase in muscle uptake of glucose (Hargreaves, 2015). A rise in glucagon and fall in insulin mobilize hepatic glycogenolysis and gluconeogenesis to supply energy to the working muscle (Trefts et al., 2015; Pillon Barcelos et al., 2017) (Figure 4). An interesting hypothesis is exercise-induced elevations in glucagon might activate CRTC2, which would increase epigenetic marks in gluconeogenic gene regions. During low-to-moderate intensity, prolonged duration exercise, there is shift in fuel utilization from carbohydrates to increased reliance on fatty acids. Fatty acids are stored in the body as triacylglycerides, which are composed of three fatty acids and a glycerol. Triacylglycerides are stored in adipocytes (as previously discussed) but also skeletal muscle. During exercise, TAGs can be broken down through lipolysis. As previously mentioned, a high rate of basal lipolysis in the context of obesity contributes to insulin resistance, but lipolysis during exercise is an important mechanism to supply fuel to working muscle. Skeletal muscle can oxidize fatty acids for ATP production and the liver uses glycerol to synthesize glucose (Figure 4). Skeletal muscle also produces large amounts of lactate, especially during high-intensity exercise, which can be converted to glucose in the liver via the Cori Cycle (Brooks, 2018) (Figure 4). The liver also helps reduce inflammation and oxidative stress during acute exercise (Pillon Barcelos et al., 2017).

Repeated bouts of exercise cause long-term liver adaptations, including enhanced hepatic gluconeogenic capabilities, decrease *de novo* lipogenesis and hepatic fat stores, and improved hepatic fatty acid oxidation (Brouwers et al., 2016; Knudsen et al., 2016; Pillon Barcelos et al., 2017). A single 90-min treadmill run decreased hepatic expression of a lipogenic transcription factor (Alex et al., 2015) and this downregulation is likely augmented over repeated bouts of exercise. Treadmill run training decreased hepatic expression of lipogenic genes (Cho et al., 2014), increased expression of oxidation-related genes (Cho et al., 2014; Alex et al., 2015) and reduced TAG levels by 30% in mice (Alex et al., 2015). Given these transcriptional changes, and evidence of genome-wide DNA methylation remodeling with exercise (Zhou et al., 2017), it is possible that epigenetic changes contribute to upregulating fatty acid oxidation genes and downregulating *de novo* lipogenesis genes. Liver adaptations to exercise involve crosstalk between skeletal muscle and WAT; but are not well-understood. Increased glucose disposal, improved insulin sensitivity, and decreased visceral fat are all likely contributing factors but the molecular mechanisms governing these adaptations (Stevanović et al., 2020), including epigenetic modifications, need to be examined. Exercise also induces hepatic mitochondrial biogenesis (mediated by the previously mentioned transcriptional co-activator pgc-1alpha) and improves mitochondria function, morphology, and bioenergetics (Stevanović et al., 2020). Epigenetic mechanisms such as SIRT7/ GABPβ1 signaling (see Liver Mitochondria section above) could also be important during exercise and contribute to mitochondrial adaptations to exercise in the liver, but this has not yet been explored.

Importantly, exercise can mitigate mitochondrial impairments in high-fat diet models of NAFLD, including NASH, and thus far weight reduction and lifestyle modifications (including exercise) are the only FDA-approved treatments for NAFLD (Stevanović et al., 2020). Aberrant DNA methylation in genes involved in lipid metabolism and mitochondrial biogenesis was found in liver biopsy samples from patients with NAFLD (Stevanović et al., 2020). Future studies are needed to determine if epigenetic modifications govern liver mitochondria adaptations to exercise and health benefits of exercise in NAFLD.

### Conclusions

This systematic review revealed evidence for metabolism-related epigenetic changes in WAT and liver, with hypothesized overlapping targets between metabolism-related and exercise-induced epigenetic changes in WAT and liver. Metabolites are necessary substrates for epigenetic modifications to occur, such as acetyl-CoA supplying histone acetylation and S-adenosyl-L-methionine supplying methylation. There are robust examinations of metabolite supply to epigenetic modifications in the cancer literature, but no direct examinations of metabolite supply to epigenetic modifications in WAT and the liver literature. While there is some evidence of acute and long-term exercise-induced changes in DNA methylation in WAT in humans, the upstream mechanisms governing these changes remain unelucidated (Ronn et al., 2013; Fabre et al., 2018). Further, exercise has been shown to prevent global DNA methylation changes associated with a fast-food diet without changing overall methyl group or methylation enzyme levels, suggesting altered methylation patterns could be due to co-factor availability (Zhou et al., 2017). Studying exercise-associated epigenetic changes in these key metabolic organs could help unravel the molecular mechanisms governing the long-term health benefits of exercise, and importantly, their role in preventing chronic diseases.

**Figure 5:**
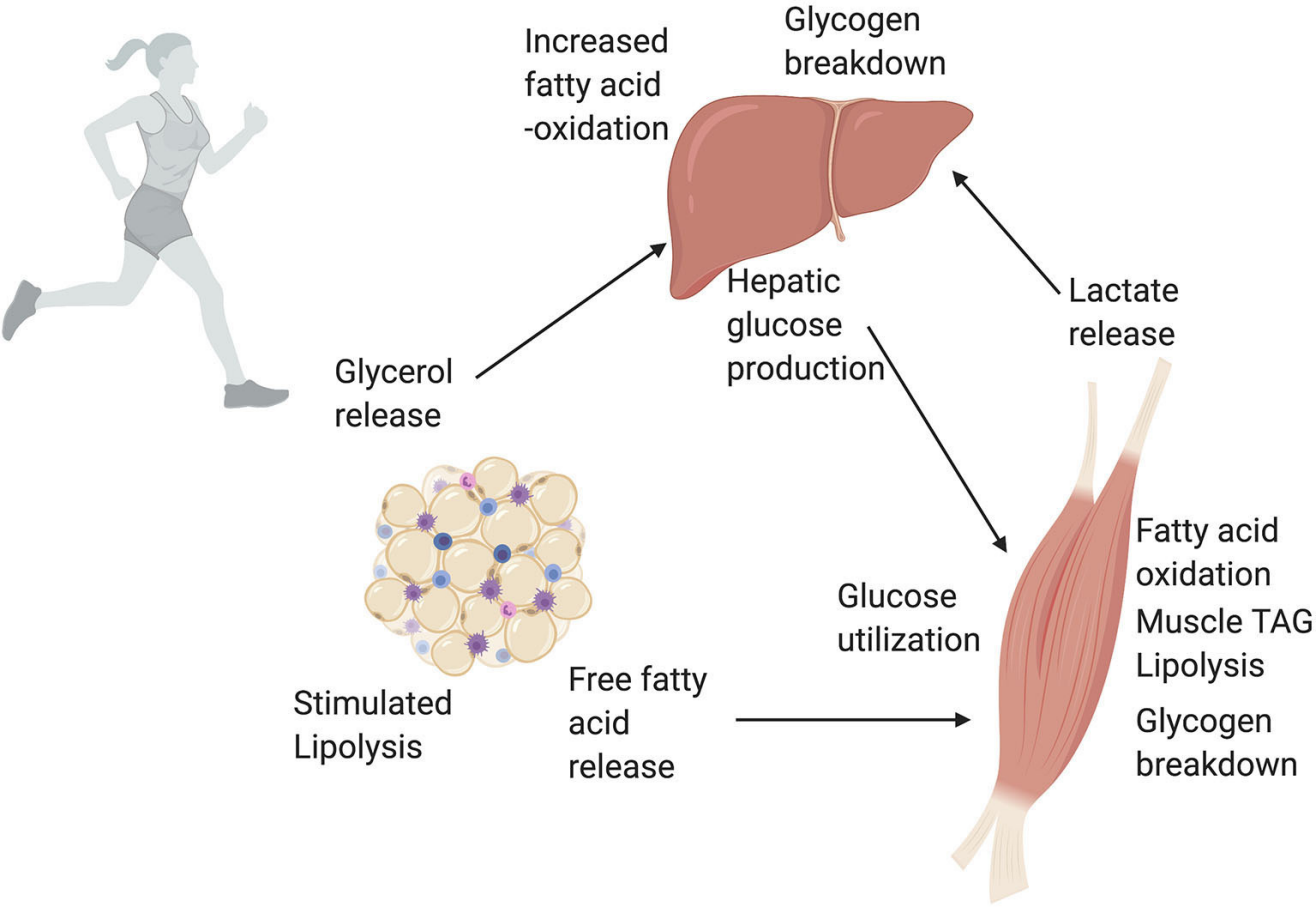
A simplified overview of white adipose tissue (WAT), liver, and skeletal muscle cross talk during exercise. Exercise massively increases the energy demands of working skeletal muscle. To meet these energy demands, skeletal muscle uses a variety of metabolic pathways depending on exercise intensity and duration. One of the largest energy suppliers during exercise is the oxidation of lipids and glucose. The rise in glucagon and fall in insulin during exercise trigger the liver to produce glucose that is then used by skeletal muscle. The increased energy demand on the liver to produce glucose is met through increased fatty acid oxidation in the liver. Additionally, lactate released via glycolysis in skeletal muscle can be converted to glucose in the liver via the Cori Cycle. An increase in circulating catecholamines and release of hormones during exercise stimulates lipolysis, or the breakdown of TAGs, in white adipose tissue. This catabolism of TAGs releases free fatty acids into the blood stream. Skeletal muscle uses these free fatty acids from the circulation, as well as lipolysis of intramuscular TAGs, for fatty acid oxidation and energy production. Finally, the glycerol released by WAT can be used by the liver to produce glucose. Through these series of networks, WAT, liver, and skeletal muscle all work together during exercise to meet increased energy demands. This figure created with Biorender.com.

Exercise demands a high level of metabolic flexibility and rapid changes in the cellular environment. White adipose tissue and the liver have important roles in both maintaining overall metabolic homeostasis at rest and helping meet the energy demands of working skeletal muscle during exercise (Figure 5). White adipose tissue lipolysis, hepatic *de novo* lipogenesis, and hepatic gluconeogenesis are key metabolic processes that demand complex transcriptional responses. The literature, while a small sample size, provided evidence that changes in DNA methylation, histone acetylation, histone methylation, and microRNA activity are important contributors to these transcriptional responses. These epigenetic modifications may likely be pertinent during exercise, when there are marked metabolic and transcriptional responses, but are best described thus far in skeletal muscle and warrant investigations in WAT and the liver (see *Future Investigations* in Table 2*)*. These investigations could provide insight into the molecular mechanisms governing long-term adaptations to exercise. Identifying and understanding exercise-induced adaptations could lead to improved exercise interventions and the potential development of new therapies for common chronic conditions, such as metabolic syndrome and fatty liver diseases. Metabolism-modulated epigenetic changes present an appealing area of future study with significant clinical relevance.

**Table 2:** Areas of future investigation to examine metabolism-related exercise-induced epigenetic changes.

**Future investigations**
**General Questions:**
- What is the upstream exercise stimulus (beta-adrenergic stimulation, hormone release) that triggers an epigenetic change?
- What are the specific epigenetic marks, what enzymes are mediating them, and what substrates are supplying the enzymes?
- How do demands for metabolic substrates during exercise affect availability for epigenetic marks?
- Do epigenetic changes correlate to changes in RNA and protein?
- How does exercise duration, frequency, and intensity affect epigenetic changes?
**Tissue-specific questions:**
Does exercise trigger epigenetic modifications that target ATGL regulation in white adipose tissue?
- What are the downstream effects on both basal and stimulated lipolysis?
Does exercise induce epigenetic modifications that upregulate fatty acid oxidation genes in white adipose tissue?
- How does this contribute to long-term white adipose tissue health?
How does the influx of fatty acids from white adipose tissue affect exercise-induced epigenetic modifications in skeletal muscle?
- Do the fatty acids contribute to histone acetylation, even when energy demands are high?
Does the rise in glucagon and fall in insulin during exercise cause epigenetic regulation of gluconeogenic genes in the liver?
- How do these changes contribute to exercise performance and overall liver health?
Do repeated bouts of exercise cause epigenetic changes that downregulate *de novo* lipogenesis genes in the liver?
- Does exercise cause the same altered methylome as dietary restriction?
- Does exercise change the expression of any of the regulatory microRNAs mentioned?
How do exercise-induced epigenetic changes affect liver mitochondria?
- What are the clinical applications of this in fatty liver diseases?
How does the influx of fatty acids from adipose tissue during exercise affect substrate usage for epigenetic changes in the liver?

*Questions for future investigations to explore are organized into general questions and tissue-specific questions related to WAT and liver*.

### Limitations

The topic of exercise-induced epigenetic modifications related to metabolism in adipose tissue and the liver is a nascent area of study. Although our literature searches from the past 10 years ensures up-to-date results, it may have limited our scope and yielded very few results. Additionally, many of the included studies relied on cell culture and mouse models. While these are important basic science approaches, the translational relevance to humans is not guaranteed. Of the studies that used mouse models, to our knowledge none have studied mice at thermoneutrality. This is an important point in metabolism research, as standard laboratory temperatures induce non-shivering thermogenesis and can affect both WAT and liver metabolism. Most included studies (except Feng et al., 2011) examined mice during their sleep cycle, and as circadian rhythms have well-established effects on metabolism, likely impacted study results and could hinder translational potential.

## Data Availability Statement

The original contributions presented in the study are included in the article/supplementary material, further inquiries can be directed to the corresponding author/s.

## Author Contributions

JEA and JRL participated in the conceptualization, study design, and writing of the manuscript. HDS and LAM participated in editing and revising the manuscript. All authors made significant contributions to this study and have approved the final version of the manuscript.

## Conflict of Interest

The authors declare that the research was conducted in the absence of any commercial or financial relationships that could be construed as a potential conflict of interest.

## Publisher's Note

All claims expressed in this article are solely those of the authors and do not necessarily represent those of their affiliated organizations, or those of the publisher, the editors and the reviewers. Any product that may be evaluated in this article, or claim that may be made by its manufacturer, is not guaranteed or endorsed by the publisher.

## Nomenclature

T2DM: Type 2 diabetes mellitus
HAT: Histone acetyltransferase
HDAC: Histone deacetyltransferase
DNMT: DNA methyltransferases
TAG: Triacylglycerides
TCA: Tricarboxylic acid
WAT: White adipose tissue
ATGL: Adipose triglyceride lipase
H3K4me3: Histone 3 lysine 4 trimethylation
AMPK: AMP-activated protein kinase
ChREBP: Carbohydrate response element binding protein
FASN: Fatty acid synthase
SCD1: Stearoyl-CoA desaturase-1
NASH: Non-alcoholic steatohepatitis
CREB: cAMP response element-binding protein
CRTC2: CREB-regulated transcriptional coactivator 2
KAT2B: Lysine acetyltransferase 2B
WDR5: WD repeat-containing protein 5
H3K9ac: Histone 3 lysine 9 acetylation
PRMT5: Arginine methyltransferase 5
H3R2me: Histone 3 arginine 2 methylation
TCF19: Transcription factor 19
NuRD: Nucleosome-remodeling deacetylase
ATF4: Activating transcription factor 4
NAFLD: Non-alcoholic fatty liver diseases
DNL: *De novo* lipogenesis
